# Chronic Groin Pain After Hernia Surgery: What Are We Missing?

**DOI:** 10.3390/jcm14176136

**Published:** 2025-08-29

**Authors:** Roberto Cirocchi, Paolo Bruzzone, Bruno Cirillo, Sara Lauricella, Gianluca Costa, Francesco Brucchi, Maria Chiara Ranucci, Massimo Lancia, Piergiorgio Fedeli, Luca Tomassini

**Affiliations:** 1 Department of Digestive and Emergency Surgery, “S. Maria” Hospital, 05100 Terni, Italy; 2 Department of Medicine and Surgery, University of Perugia, 06129 Perugia, Italy; massimo.lancia@unipg.it; 3 Department of General and Specialist Surgery, University of Roma La Sapienza, 00185 Rome, Italy; paolo.bruzzone@uniroma1.it; 4 Department of General Surgery, IRCCS Multimedica, 20099 Milan, Italy; bruno.cirillo@uniroma1.it; 5 Colorectal Surgery Unit, Fondazione IRCCS Istituto Nazionale Dei Tumori, 20133 Milan, Italy; lauricella3008@gmail.com; 6 Surgery Center, Colorectal Surgery Research Unit, Fondazione Policlinico Universitario Campus Bio-Medico, University Campus Bio-Medico of Rome, 00128 Rome, Italy; g.costa@unilink.it; 7 General Surgery, Department of Life Sciences, Health, and Health Professions, Link Campus University, 00165 Rome, Italy; 8 General Surgery Residency Program, University of Milan, 20122 Milan, Italy; francesco.brucchi@unimi.it; 9 Division of Surgery, Istituto Auxologico Italiano, Istituto di Ricovero e Cura a Carattere Scientifico IRCCS, 20122 Milan, Italy; 10 Division of Surgery, Hospital Leopoldo Parodi Delfino, 00034 Colleferro, Italy; ranucci.c@gmail.com; 11 School of Law, Legal Medicine, University of Camerino, 62032 Camerino, Italy; piergiorgio.fedeli@unica.it; 12 School of Advanced Studies, University of Camerino, 62032 Camerino, Italy; luca.tomassini@unicam.it

**Keywords:** chronic postoperative inguinal pain [CPIP], inguinal hernia repair, laparoscopic hernia repair, open hernia repair, neuropathic pain, nociceptive pain, mesh-related complications, pain assessment tools, surgical outcomes

## Abstract

**Background:** Chronic postoperative inguinal pain [CPIP] is a prevalent and often debilitating complication following inguinal hernia repair. With the widespread adoption of mesh-based techniques, recurrence rates have significantly declined, shifting clinical focus toward postoperative pain management. **Methods:** This narrative review synthesizes international literature on CPIP incidence, surgical technique, geographic variation, and the distinction between neuropathic and nociceptive pain. Studies were selected based on relevance, sample size, and inclusion of pain subclassification. **Results:** CPIP incidence varies markedly across studies (6–64.3%), influenced by follow-up duration, surgical approach, and regional healthcare practices. The risk of CPIP varies significantly, depending on the surgical technique employed, with open repairs generally associated with higher rates than laparoscopic approaches. Neuropathic pain predominates in specific cohorts, particularly following open repairs with limited nerve preservation. Few studies differentiate pain types, revealing a critical gap in diagnostic precision. **Conclusions:** CPIP is a multifactorial and under-recognized problem in clinical practice. The adoption of standardized diagnostic tools and long-term follow-up protocols is essential to improve pain classification and management. A structured diagnostic algorithm may assist clinicians in distinguishing pain types and tailoring treatment strategies to individual patient profiles.

## 1. Introduction

Inguinal hernia repair is among the most frequently performed surgical procedures worldwide, with millions of cases annually. The widespread adoption of mesh-based techniques has led to a substantial reduction in recurrence rates—estimated at between 50% and 75% [1]. Consequently, clinical attention has shifted from recurrence prevention to the management of postoperative complications, particularly chronic groin pain.

Several surgical techniques are currently employed for inguinal hernia repair. Open procedures, such as Lichtenstein, Shouldice, and Bassini, remain widely used but carry risks of nerve injury and mesh-related inflammation [2]. Minimally invasive approaches, including Trans Abdominal Pre Peritoneal (TAPP) and Totally Extra Peritoneal (TEP) laparoscopy, are associated with faster recovery and, in most series, lower rates of chronic pain, although specific complications may occur [3,4]. Robotic-assisted repair, a more recent innovation, offers enhanced precision and ergonomics, but current evidence remains limited [5,6,7]. Each technique has its own risk profile, and postoperative pain has emerged as one of the most relevant complications, sometimes persisting beyond the expected recovery period.

While post-herniorrhaphy discomfort typically resolves within two months [2], a subset of patients continues to experience persistent pain beyond this period. This condition is known as chronic postoperative inguinal pain (CPIP), also referred to in pain literature as Persistent Postsurgical Pain (PPSP) after inguinal hernia repair [3,4]. Originally defined by the International Association for the Study of Pain (IASP) in 1986 as pain persisting for more than three months after surgery, CPIP was later refined by the HerniaSurge Group [2018] [7]. The updated definition includes pain that is moderate to severe, lasts beyond three months, and interferes with daily activities such as movement, sleep, or social interaction [5,6,7]. However, due to ongoing mesh-related inflammation beyond the three-month period, some experts advocate extending the diagnostic threshold to six months [8].

Recent studies have further expanded the understanding of CPIP, highlighting the importance of individualized surgical strategies [9]. Huy et al. (2025) demonstrated that tailored interventions, including neurectomy and mesh removal, can significantly improve outcomes in patients with refractory CPIP [9]. Kwee et al. (2024) emphasized the role of selective and triple neurectomy in neuropathic CPIP, reporting pain relief in over two-thirds of treated patients [10]. Moreover, Reichert et al. (2025) investigated the inflammatory profiles of different mesh types, suggesting that material selection may influence long-term pain outcomes [11]. Li et al. (2025) compared robotic and laparoscopic techniques, showing that robotic-assisted repair may reduce reoperation rates and enhance postoperative recovery, although further validation is needed [12].

Despite consensus definitions, the diagnostic threshold remains debated, particularly in cases of prolonged mesh-related inflammation [13]. A review of the literature offers a comprehensive overview of CPIP following inguinal hernioplasty, across various studies and geographic regions (Table 1), with incidence rates ranging from 6% to 64.3% across different countries and surgical approaches [13,14,15,16,17,18,19,20,21,22,23,24,25,26,27,28,29,30,31,32]. Importantly, the risk of chronic postoperative inguinal pain (CPIP) is not uniform across all hernia repair techniques. Open mesh-based procedures, such as Lichtenstein or Shouldice, tend to show higher CPIP rates due to increased nerve exposure and mesh fixation [33]. In contrast, laparoscopic approaches like TEP and TAPP generally yield lower pain incidence, although exceptions exist and technique-specific complications may arise [34]. Robotic-assisted repairs, while promising, remain under investigated [35]. Therefore, any analysis of CPIP must be contextualized within the specific surgical technique employed, to avoid misleading associations and ensure accurate interpretation of outcomes [36].

## 2. Methodology

This narrative review was conducted through a comprehensive literature search using PubMed, Scopus, and Web of Science databases. Keywords included “chronic postoperative inguinal pain”, “CPIP”, “Persistent Postsurgical Pain”, “hernia repair”, “neuropathic pain”, “nociceptive pain”, and “neurectomy”. Studies published between January 2000 and April 2025 were considered.

Inclusion criteria were the following: sample size ≥100 patients, clear subclassification of pain types, relevance to surgical technique and postoperative pain outcomes, and full-text availability in English. Exclusion criteria included case reports, editorials, and studies lacking pain classification or follow-up data. Reference lists of selected articles were manually screened to identify additional relevant studies.

## 3. Epidemiological Landscape

The international literature on CPIP reveals a complex and heterogeneous picture. Incidence rates vary widely—from 6% in Forester’s UK-based study (2021) to an exceptional 64.3% in Niebuhr’s German cohort (2018)—highlighting the multifactorial nature of CPIP [15,22]. This variability reflects a dynamic interplay of factors, including surgical technique, follow-up duration, patient selection criteria, regional healthcare practices, and methodological consistency.

A clear trend emerges regarding follow-up duration. Studies with shorter postoperative evaluations [3–6 months], such as Lo (2021: 8.7%) and Forester [2021: 6%], tend to report lower CPIP rates [14,15]. In contrast, studies with extended follow-up periods [≥12 months], including Lundström (2018: 15.2%) and Jeroukhimov (2014: 32.8%), consistently report higher pain prevalence [20,29]. This pattern suggests that early assessments may underestimate the true burden of chronic pain, reinforcing the importance of long-term surveillance in clinical research. Recent evidence supports this view. Gutlic et al. (2024) conducted an 8-year follow-up comparing TEP and Lichtenstein repairs, showing that CPIP rates remain stable over time and may persist well beyond the first postoperative year [40]. Narita et al. (2024) reported that 88% of patients undergoing surgery for CPIP experienced excellent or good outcomes after a median of 24.6 months, reinforcing the need for extended monitoring and individualized treatment strategies [41]. Chu et al. (2024) performed a meta-analysis of 29,466 patients and found a pooled CPIP incidence of 17.01%, with higher rates in Europe (18.65%) compared to Asia (14.70%) and North America (6.04%), highlighting geographic variability and the importance of regional surveillance protocols [42]. These findings underscore the need for standardized long-term follow-up protocols to accurately assess CPIP prevalence and guide postoperative care.

## 4. Surgical Technique and Geographic Variation

Surgical technique plays a pivotal role in CPIP outcomes. Laparoscopic repairs generally yield lower CPIP rates compared to open approaches. For example, Lo (2021) and Gutlic (2019) report rates below 9% in laparoscopic cohorts, supporting the hypothesis that minimally invasive techniques may reduce nerve trauma and mesh-related inflammation [14,27]. However, this advantage is not absolute. Niebuhr’s 2018 study presents a striking anomaly: a CPIP rate of 64.3%, despite exclusive use of laparoscopy, with a notably large sample size of 20,004 patients [22]. This outlier raises methodological concerns, and suggests the influence of confounding variables such as non-standardized surgical protocols, selection bias, or inconsistencies in pain assessment. It serves as a reminder that no technique is inherently superior without rigorous execution and individualized patient care.

A recent study by Liu et al. (2025) further supports this perspective, reporting significantly higher chronic pain rates in the open repair group compared to the laparoscopic group (4.8% vs. 1.88%, *p* < 0.05) [43]. Similarly, in a prospective cohort by Thulasilingam et al. (2023), patients undergoing laparoscopic TAPP repair experienced significantly lower pain scores and faster return to activity, compared to those receiving open mesh repairs, reinforcing the role of technique in postoperative recovery [44]. A randomized controlled trial by Ulutaş and Yilmaz (2025) comparing laparoscopic TEP and open Lichtenstein repair in elderly patients found substantially lower chronic pain rates in the laparoscopic group (1.7% vs. 10%, *p* = 0.05), along with faster recovery and reduced analgesic use [45].

The interpretation of CPIP incidence must be contextualized within the specific surgical technique employed. As shown in Table 1, studies reporting CPIP outcomes vary in their use of open, laparoscopic, or mixed approaches, each of which significantly influences postoperative pain profiles. Open mesh-based repairs, such as the Lichtenstein technique, are generally associated with higher rates of chronic pain, due to increased nerve exposure and fixation methods. In contrast, laparoscopic techniques like TEP and TAPP tend to yield lower CPIP rates, likely due to reduced tissue dissection and minimized nerve handling. Nevertheless, exceptions exist—such as the elevated pain rates observed in certain laparoscopic cohorts—which underscore the importance of surgical precision, adherence to standardized protocols, and individualized patient care. Therefore, any analysis of CPIP must carefully distinguish between surgical modalities, to avoid misleading associations and ensure accurate clinical interpretation.

Geographic variation further complicates the CPIP landscape. European studies often report moderate-to-high CPIP rates—Fränneby (2006, Sweden): 31% [38], Nienhuijs (2005, Netherlands): 43.3% [39] suggest chronic postoperative pain may affect up to 30% of patients across various procedures, highlighting the multifactorial nature of pain outcomes [3]. In contrast, Asian studies such as Lo (2021, Taiwan) [14] and Min (2020, China: 26.8%) [16] tend to report lower or intermediate rates, raising questions about regional differences in clinical practice, mesh selection, perioperative care, and even genetic predisposition to chronic pain. A recent meta-analysis by Chu et al. (2024) confirmed this trend, showing CPIP rates of 18.65% in Europe, 14.70% in Asia, and 6.04% in North America across 29,466 patients [42].

These discrepancies indicate that CPIP is not solely a surgical issue, but also reflects broader systemic and cultural determinants. Interestingly, surgical expertise does not appear to significantly influence CPIP rates. As demonstrated by de la Croix (2025), the incidence was nearly identical between patients operated on by specialist surgeons and those treated by surgical residents (15.4% vs. 15.5%) [46].

This finding aligns with the results of Lange et al. (2016), who found no statistically significant difference in CPIP rates between expert and non-expert surgeons in Lichtenstein repairs, despite a trend toward better outcomes in high-volume operators [47].

## 5. Strengths and Limitations of the Literature

From a critical standpoint, the dataset presents several strengths. It encompasses a wide range of countries, surgical techniques, and follow-up durations, offering a broad overview of CPIP across diverse clinical settings. The inclusion of large patient samples—such as Niebuhr’s 2018 cohort of over 20,000 individuals—enhances the statistical reliability of reported outcomes [22]. Recent multicenter studies, such as Liu et al. (2025) and Chu et al. (2024), have further expanded the evidence base by incorporating diverse geographic cohorts and standardized pain metrics, improving external validity and comparative potential [42,43]. However, limitations are equally evident. The heterogeneity in study designs—particularly regarding follow-up intervals and CPIP definitions—complicates direct comparisons. The presence of “NR” (Not Reported) values in several studies further obscures the timeline of pain evaluation, while outlier data—such as the unexpectedly high CPIP rate in Niebuhr’s laparoscopic series—requires deeper methodological scrutiny to identify potential sources of bias or inconsistency.

Clinically, these findings underscore the urgent need for standardized pain assessment tools to improve data consistency and comparability. Instruments such as the DN4 questionnaire should be routinely employed to distinguish between neuropathic and nociceptive pain, thereby refining diagnostic accuracy. A 2025 systematic review by Madani et al. confirmed the DN4’s strong psychometric properties, with pooled sensitivity of 82% and specificity of 76.1%, supporting its use in chronic pain cohorts, including CPIP [48]. Hardt et al. (2023) validated the English 7-item DN4 version, demonstrating excellent short- and long-term test–retest reliability (weighted kappa > 0.85), reinforcing its applicability in longitudinal CPIP studies [49]. Although laparoscopic approaches generally appear to yield better outcomes in terms of CPIP reduction, the presence of elevated pain rates in certain laparoscopic cohorts highlights the need for further investigation into surgical technique, perioperative management, and patient-specific variables. Moreover, regional differences in CPIP prevalence should be explored in greater depth, to develop tailored pain prevention strategies that align with local healthcare infrastructure and demographic profiles.

Chu et al. (2024) highlighted significant geographic variation, with CPIP rates of 18.65% in Europe, 14.70% in Asia, and 6.04% in North America, suggesting that cultural, systemic, and clinical factors play a substantial role in pain outcomes [42].

Future research should prioritize harmonization of study designs and the adoption of validated pain assessment tools to enable meaningful comparisons and guide evidence-based interventions. Routine use of instruments such as the DN4 questionnaire should become standard practice in both research and clinical settings.

## 6. Neuropathic vs. Nociceptive CPIP: Diagnostic Challenges and Clinical Implications

Understanding the nature of CPIP is essential for effective treatment. Differentiating between neuropathic and nociceptive pain is not merely academic—it directly influences therapeutic choices. In routine clinical practice, distinguishing between neuropathic and non-neuropathic CPIP remains a significant challenge. Although the underlying mechanisms differ, their clinical manifestations often overlap, making accurate classification difficult [10,50]. A re-analysis of selected studies reporting CPIP incidence reveals that only a limited number of authors have attempted to differentiate between neuropathic and nociceptive groin pain—highlighting a significant gap in diagnostic precision (Table 2).

Recent evidence confirms this diagnostic gap. Kwee et al. (2024) conducted a systematic review of surgical treatments for neuropathic CPIP, and found that only 10 studies explicitly classified pain type prior to intervention, underscoring the lack of standardized diagnostic frameworks [10]. Hardt et al. (2023) validated the English version of the DN4 questionnaire, showing excellent reliability (weighted kappa > 0.85), and recommended its routine use in CPIP studies to distinguish neuropathic components [49]. Nijs et al. (2023) emphasized the importance of pain phenotyping in chronic groin pain, proposing a stratified approach based on predominant pain type—nociceptive, neuropathic, or nociplastic—to guide individualized treatment [51].

Most of these studies focus on open surgical techniques, with only Loos (2007) including laparoscopic procedures [37]. The inclusion of laparoscopy in this cohort may influence CPIP outcomes, potentially shifting the balance between neuropathic and nociceptive pain.

The absence of consistent pain subclassification across studies limits the development of targeted treatment strategies. A standardized diagnostic framework is urgently needed, to improve patient outcomes.

Van Veenendaal et al. (2023) highlighted the fact that most non-surgical CPIP studies fail to report pain phenotype, making it difficult to assess treatment efficacy and select appropriate interventions [52]. Emerging approaches such as targeted muscle reinnervation (TMR) show promise in neuropathic CPIP, but require precise preoperative pain classification, to optimize outcomes [10].

## 7. Neuropathic and Nociceptive Pain Profiles

Neuropathic CPIP rates vary widely. Ergönenç (2017) reports the highest proportion [73.7%], suggesting a predominant neuropathic component likely associated with limited nerve preservation during open repair [23]. Bande (2020) and Nienhuijs (2005) report similar rates (38.5% and 40.3%, respectively), possibly reflecting comparable surgical techniques or perioperative protocols [13,39]. Loos (2007), with a rate of 46.5%, occupies a mid-range position—potentially influenced by the inclusion of laparoscopic procedures, which may mitigate nerve trauma [37].

Recent evidence from Kwee et al. (2024) confirms the predominance of neuropathic pain in CPIP, with pooled estimates showing neuropathic features in up to 68% of patients undergoing surgical treatment for refractory groin pain [10].

Charitakis et al. (2025) reported that triple neurectomy yielded pain improvement in 98.2% of neuropathic CPIP cases, reinforcing the importance of accurate pain classification in surgical decision-making [53].

Conversely, non-neuropathic CPIP rates show an inverse trend. Ergönenç (2017) reports the lowest rate (26.3%), while Bande (2020) and Nienhuijs (2005) report higher rates (61.5% and 59.7%) [13,23,39]. These discrepancies may be attributed to variations in pain classification criteria, diagnostic methodology, or postoperative nerve handling. Van Veenendaal et al. (2023) emphasized that most non-surgical CPIP studies fail to report pain phenotype, making it difficult to assess treatment efficacy and select appropriate interventions [52]. The relatively balanced distribution in Loos (2007) further supports the hypothesis that surgical approach—particularly laparoscopy—may influence the type of pain experienced [37].

These findings are consistent with the broader framework of Persistent Postsurgical Pain (PPSP), which integrates both neuropathic and nociceptive components and is increasingly recognized in the pain literature as a distinct clinical entity.

Fuller et al. (2023) described PPSP as affecting 10–50% of surgical patients, with central sensitization and neuroimmune mechanisms contributing to chronicity, especially in procedures involving nerve-rich regions like the groin [54]. Moka et al. (2024) highlighted the need for transitional pain services and early phenotyping to prevent PPSP evolution, particularly in high-risk surgeries such as hernia repair [55].

## 8. Limitations and Methodological Considerations

Several limitations must be acknowledged. Sample sizes vary significantly—from 61 patients in Ergönenç (2017) to 239 in Bande (2020)—which may affect the statistical reliability and generalizability of findings [13,23]. Latenstein et al. (2021) highlighted substantial hospital-level variation in inguinal hernia repair practices, suggesting that surgical outcomes may be influenced by institutional preferences rather than standardized evidence-based protocols [56].

Additionally, the lack of precise differentiation between open techniques (e.g., Lichtenstein vs. Shouldice), as well as between minimally invasive approaches such as TAPP (Transabdominal Preperitoneal) and TEP (Totally Extraperitoneal), may obscure subtle trends and hinder direct comparisons across surgical modalities [43].

Henriksen et al. (2024) emphasized the fact that laparoscopic techniques, while technically demanding, are associated with lower surgical-site infection rates and improved postoperative quality of life, though they require greater surgical expertise [57]. Crepaz et al. (2025), in the ACTIVE study, demonstrated that laparoscopic approaches in emergency settings resulted in fewer complications and shorter hospital stays, compared to open surgery [58].

Inconsistencies in pain classification—particularly in distinguishing neuropathic from nociceptive pain—further limit cross-study comparability.

Pedersen et al. (2021) proposed a simplified clinical algorithm for diagnosing and surgically treating chronic post-herniorrhaphy pain, incorporating tools such as PainDETECT and Quantitative Sensory Testing (QST) [59]. Van Veenendaal et al. (2023) noted that most non-surgical CPIP studies fail to report pain phenotype, limiting the ability to assess treatment efficacy and select appropriate interventions [52].

From a clinical standpoint, the high prevalence of neuropathic pain in Ergönenç (2017) underscores the importance of meticulous nerve identification and preservation during hernia repair [23]. The potential protective effect of laparoscopy, as suggested by Loos (2007), merits further investigation, to clarify whether minimally invasive techniques reduce the risk of neuropathic complications [37].

Sun et al. (2025) found that laparoscopic obturator hernia repair significantly reduced systemic inflammation and hospital stay duration compared to open surgery, suggesting a broader benefit in postoperative pain outcomes [60].

Ultimately, pain management strategies should be tailored to individual patient risk profiles, with careful consideration of surgical technique, intraoperative nerve handling, and structured postoperative monitoring.

Jensen et al. (2025) initiated the STRONG-Hernia trial, evaluating the impact of personalized prehabilitation programs on postoperative pain and complications in patients with modifiable risk factors [61]. Alzatari et al. (2024) demonstrated that non-opioid analgesic regimens were non-inferior to opioid-based protocols in managing post-herniorrhaphy pain, supporting a safer multimodal approach [62].

## 9. Toward a Structured Diagnostic Approach

These findings offer valuable insights into the relative prevalence of neuropathic pain among CPIP cases. Notably, Ergönenç (2017) reported a striking 73.7% incidence of neuropathic CPIP in an open repair cohort, suggesting nerve injury as a predominant mechanism in certain surgical contexts [23]. Other studies show a more balanced distribution, indicating that both neuropathic and nociceptive pathways contribute meaningfully to postoperative pain.

Recent evidence confirms this dual-pathway model. Charitakis et al. (2025) found that triple neurectomy led to pain improvement in over 98% of patients with neuropathic CPIP, while double neurectomy offered complete relief in 80% of cases, albeit with a higher complication rate [53]. Kwee et al. (2024) reported that targeted muscle reinnervation yielded the highest success rate among surgical techniques for refractory neuropathic CPIP, with 88% of patients experiencing significant pain relief [10].

Despite these observations, the limited number of studies performing this subclassification highlights a critical gap in the literature—Van Veenendaal et al. (2023) emphasized that most non-surgical CPIP studies fail to report pain phenotype, limiting treatment personalization and outcome assessment [52]. Wang et al. (2023) demonstrated that CXCL13/CXCR5 signaling contributes to mechanical allodynia and neuroinflammation in CPIP models, suggesting a molecular basis for nociceptive pain persistence [63].

In the absence of consistent use of validated diagnostic tools and standardized definitions, the true burden of neuropathic CPIP remains difficult to quantify. Further research is essential, to elucidate risk factors, refine diagnostic criteria, and optimize surgical techniques, to minimize both forms of chronic pain.

**Figure 1:**
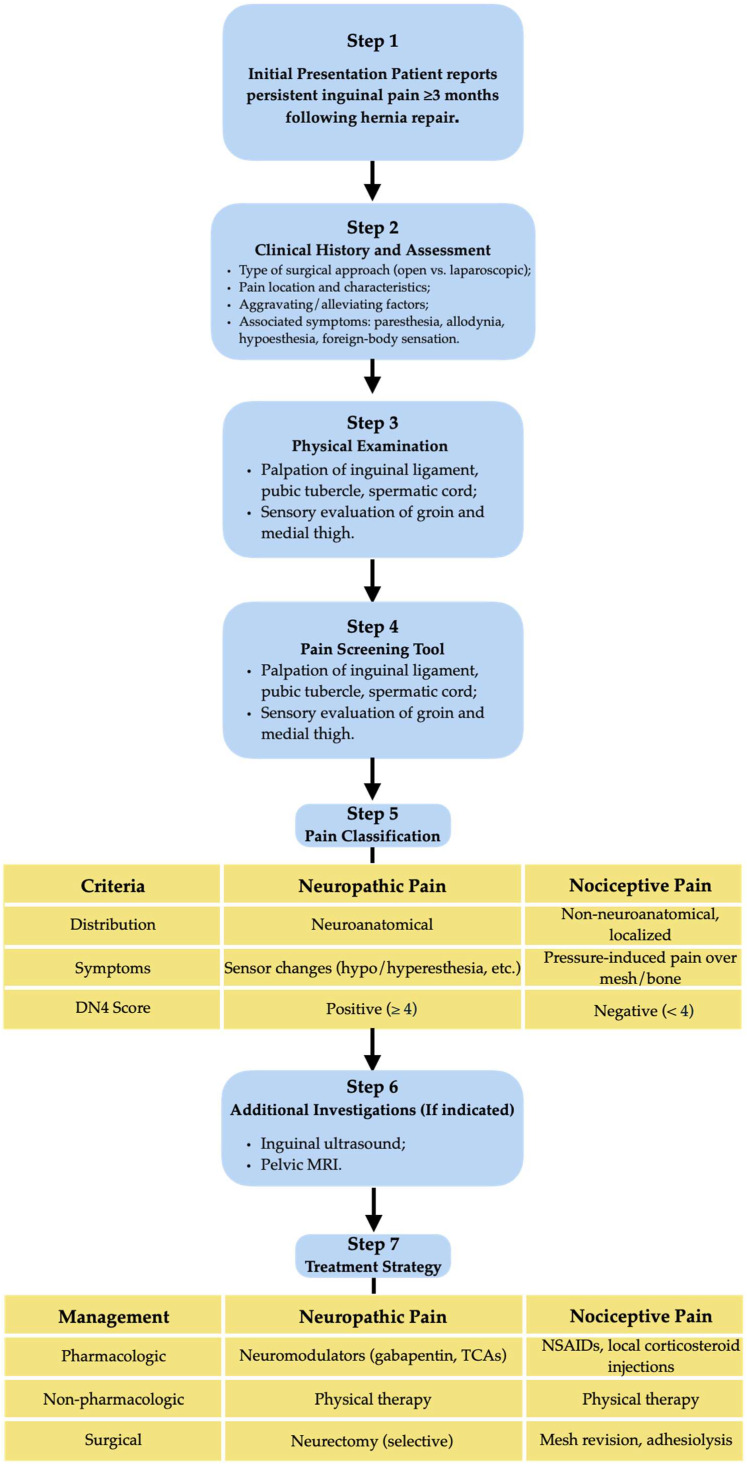
Proposed diagnostic algorithm for the assessment and management of CPIP, differentiating neuropathic and nociceptive pain.

Given the diagnostic complexity and multifactorial nature of CPIP, a structured clinical algorithm may assist surgeons and pain specialists in navigating evaluation and treatment. Pedersen et al. (2021) proposed a simplified algorithm integrating PainDETECT and Quantitative Sensory Testing (QST) to distinguish pain types and guide surgical decision-making [59]. Pawlak et al. (2022) advocated for multidisciplinary team (MDT) clinics to personalize CPIP management, especially in cases with overlapping pain mechanisms [64]. Such a pathway should integrate current evidence and clinical reasoning to distinguish between pain types, guide appropriate investigations, and tailor management strategies to individual patient profiles. This approach emphasizes early recognition, standardized assessment tools, and a stepwise therapeutic plan aimed at minimizing long-term morbidity (Figure 1).

Validation of this algorithm through prospective studies and its incorporation into clinical guidelines could significantly enhance CPIP management and reduce long-term disability.

## 10. Neurectomy in CPIP Management

Neurectomy—particularly during open inguinal hernia repair—has been proposed as both a preventive and therapeutic strategy for chronic postoperative inguinal pain (CPIP). Selective neurectomy involves intentional transection of one or more inguinal nerves (e.g., ilioinguinal, iliohypogastric, or genitofemoral) to mitigate the risk of postoperative neuropathic pain.

The technique of triple neurectomy, although more aggressive, has shown promising results in specific patient cohorts, with significant reductions in pain scores, and improvements in quality of life. In a multicenter study by Charitakis et al., 98.2% of patients undergoing triple neurectomy reported substantial clinical improvement [53].

Kwee et al. (2024) confirmed that targeted muscle reinnervation yielded the highest success rate among surgical techniques for refractory neuropathic CPIP, with 88% of patients reporting significant pain relief [10]. Moore et al. (2025) demonstrated that retroperitoneal laparoscopic triple neurectomy led to durable pain reduction and improved sensory thresholds over a 3-year follow-up, supporting its long-term efficacy in selected patients [65].

However, the procedure remains controversial. While some studies report favorable outcomes, others highlight potential complications such as sensory loss, neuroma formation, and chronic dysesthesia [10]. Cirocchi et al. conducted a meta-analysis of 16 RCTs involving 1550 patients, and found a significant reduction in groin pain at 6 months in the neurectomy group, but no clear long-term advantage over nerve preservation [66]. This aligns with findings from Singh et al. (2024), who also noted short-term benefits but questioned the durability of prophylactic neurectomy outcomes [67].

The decision to perform neurectomy should be individualized, based on intraoperative findings (e.g., nerve entrapment or inflammation), patient risk factors, and surgeon expertise. Recently, Messias et al. proposed ten recommendations to optimize outcomes in Lichtenstein hernia repair, including pragmatic neurectomy as a strategy to reduce chronic postoperative pain in selected patients [68].

Recent guidelines suggest that prophylactic neurectomy may be considered in high-risk patients undergoing open repair, particularly when nerve preservation is not technically feasible [6,7,69]. Conversely, therapeutic neurectomy should be reserved for patients with refractory neuropathic CPIP unresponsive to conservative measures [70].

Minimally invasive approaches such as laparoscopic triple neurectomy are emerging as viable options. A case report by Castillo et al. documented complete pain resolution following laparoscopic triple neurectomy, opening new avenues for CPIP treatment [71].

Further research is needed to standardize indications, refine surgical techniques, and evaluate long-term outcomes of neurectomy in CPIP management.

## 11. Conclusions

Chronic postoperative inguinal pain (CPIP) remains a clinically significant and multifactorial complication of hernia surgery. The available evidence highlights substantial heterogeneity in reported incidence, largely attributable to variations in diagnostic criteria, follow-up protocols, and methodological design. Despite these inconsistencies, a consistent finding is that CPIP often encompasses both neuropathic and nociceptive components, underscoring the importance of accurate subclassification in clinical assessment. Minimally invasive approaches are generally associated with lower pain rates, although this advantage is not universal and must be interpreted with caution. What clearly emerges from the literature is the urgent need for standardized diagnostic definitions, validated assessment tools, and long-term prospective studies. These elements are essential to enable reliable comparisons and to support the development of tailored management strategies. Advancing in this direction is crucial to reduce the burden of CPIP and improve long-term outcomes and quality of life for patients undergoing inguinal hernia repair.

## Figures and Tables

**Table 1:** Incidence of chronic postoperative inguinal pain (CPIP) across international studies by surgical approach, follow-up duration, and geographic region.

Author and Year of Publication	Nation	Time of Evaluation	Surgical Approach	Number of Patients Enrolled	Number of Patients with CPIP	Rate of Patients with CPIP
Lo et al., 2021 [14]	Taiwan	3 months	Laparoscopy (TAPP)	664	53	8.7%
Forester et al., 2021 [15]	UK	6 months	Laparoscopy (TEP)	960	58	6%
Min et al., 2020 [16]	China	3 months	Mixed (Open + TAPP)	800	215	26.8%
Bande et al., 2020 [13]	Spain	4 months	Open (Lichtenstein)	1.761	239	13.6%
Köckerling et al., 2019 [17]	Germany	1 year	Mixed (Registry Data)	15.601	1.189	7.6%
Chinchilla Hermida et al., 2017 [18]	Colombia	6 months	Mixed (Open + Laparoscopic)	108	30	27.8%
Andercou et al., 2018 [19]	Romania	3 months	Mixed (Open + Laparoscopic)	365	38	10.4%
Lundström et al., 2018 [20]	Sweden	1 year	Mixed (Open + Laparoscopic)	22.917	3.492	15.2%
Matikainen et al., 2018 [21]	Finland	1 year	Open (Lichtenstein)	625	52	8.32%
Niebuhr et al., 2018 [22]	Germany	1 year	Laparoscopic (TAPP)	20.004	12.866	64.3%
Ergönenç et al., 2017 [23]	Turkey	3 months	Open (Lichtenstein)	264	61	23.4%
Olsson et al., 2017 [24]	Sweden	NR	Open (Lichtenstein)	952	170	17.8%
Andresen et al., 2017 [25]	Denmark	NR	Laparoscopic (TEP)	1.421	278	19.5%
Pierides et al., 2016 [26]	Finland	1 year	Open (Mesh-based)	932	99	11.5%
Gutlic et al., 2016 [27]	Sweden	NR	Laparoscopic (TEP)	1.098	85	7.7%
Langeveld et al., 2015 [28]	The Netherlands	1 year	Mixed (Open + Laparoscopic)	489	130	27%
Jeroukhimov et al., 2014 [29]	Israel	1 year	Open (Mesh-based)	192	63	32.8%
Nikkolo et al., 2012 [30]	Estonia	3 years	Open (Mesh-based)	116	27	23.3%
Reinpold et al., 2011 [31]	Germany	6 months	Open (Mesh-based)	704	116	16.6%
Hompes et al., 2008 [32]	Belgium	1 year	Open (Kugel technique)	377	57	15.1%
Poobalan et al., 2003 [3]	UK	3 months	Open (Mesh-based)	226	67	30%
Loos et al., 2007 [37]	The Netherlands	NR	Mixed (Open + Laparoscopic)	1.776	211	11.9%
Fränneby et al., 2006 [38]	Sweden	NR	Open (Mesh-based)	2.456	758	31%
Nienhuijs et al., 2005 [39]	The Netherlands	NR	Open (Mesh-based)	319	139	43.3%

NR: not reported.

**Table 2:** Distribution of neuropathic and non-neuropathic CPIP by surgical technique and study cohort.

Author and Year of Publication	Number of Patients with CPIP	Surgical Approach	Patients with Neuropathic CPIP	Rate of Neuropathic CPIP	Patients with Non-Neuropathic CPIP	Rate of Non-Neuropathic CPIP
Bande 2020 [13]	239	Open	92	38.5%	147	61.5%
Ergönenç 2017 [23]	61	Open	45	73.7%	16	26.3%
Loos 2007 [37]	148	Open/Laparoscopy	72	46.5%	76	53.5%
Nienhuijs 2005 [39]	139	Open	56	40.3%	82	59.7%

## Data Availability

No new data were created or analyzed in this study. Data sharing is not applicable to this article.

## Abbreviations

The following abbreviations are used in this manuscript:

CPIP	Chronic postoperative inguinal pain
IASP	International Association for the Study of Pain
NR	Not Reported
DN4	Douleur Neuropathique 4
TAPP	Transabdominal Preperitoneal
TEP	Totally Extraperitoneal
CI	Confidence Interval
PMID	PubMed Identifier
